# Magnolol Alleviates Inflammatory Responses and Lipid Accumulation by AMP-Activated Protein Kinase-Dependent Peroxisome Proliferator-Activated Receptor α Activation

**DOI:** 10.3389/fimmu.2018.00147

**Published:** 2018-02-05

**Authors:** Ye Tian, Haihua Feng, Lu Han, Lin Wu, Hongming Lv, Bingyu Shen, Zheng Li, Qiaoling Zhang, Guowen Liu

**Affiliations:** ^1^Key Laboratory of Zoonosis, Ministry of Education, College of Veterinary Medicine, Jilin University, Changchun, China

**Keywords:** magnolol, hyperlipidemia, steatosis, AMPK, peroxisome proliferator-activated receptor α, inflammatory responses

## Abstract

Magnolol (MG) is a kind of lignin isolated from *Magnolia officinalis*, which serves several different biological functions, such as antifungal, anticancer, antioxidant, and hepatoprotective functions. This study aimed to evaluate the protective effect of MG against oleic acid (OA)-induced hepatic steatosis and inflammatory damage in HepG2 cells and in a tyloxapol (Ty)-induced hyperlipidemia mouse model. Our findings indicated that MG can effectively inhibit OA-stimulated tumor necrosis factor α (TNF-α) secretion, reactive oxygen species generation, and triglyceride (TG) accumulation. Further study manifested that MG significantly suppressed OA-activated mitogen-activated protein kinase (MAPK) and nuclear factor-kappa B (NF-κB) signaling pathways and that these inflammatory responses can be negated by pretreatment with inhibitors of extracellular regulated protein kinase and c-Jun N-terminal kinase (U0126 and SP600125, respectively). In addition, MG dramatically upregulated peroxisome proliferator-activated receptor α (PPARα) translocation and reduced sterol regulatory element-binding protein 1c (SREBP-1c) protein synthesis and excretion, both of which are dependent upon the phosphorylation of adenosine monophosphate (AMP)-activated protein kinase (AMPK), acetyl-CoA carboxylase, and AKT kinase (AKT). However, MG suspended the activation of PPARα expression and was thus blocked by pretreatment with LY294002 and compound c (specific inhibitors of AKT and AMPK). Furthermore, MG clearly alleviated serum TG and total cholesterol release; upregulated AKT, AMPK, and PPARα expression; suppressed SREBP-1c generation; and alleviated hepatic steatosis and dyslipidemia in Ty-induced hyperlipidemia mice. Taken together, these results suggest that MG exerts protective effects against steatosis, hyperlipidemia, and the underlying mechanism, which may be closely associated with AKT/AMPK/PPARα activation and MAPK/NF-κB/SREBP-1c inhibition.

## Introduction

Non-alcoholic fatty liver disease (NAFLD) is defined by the presence of hepatic steatosis, or the accumulation of excessive fat deposition in liver cells, which is often related to insulin resistance, chronic over-nutrition, and metabolic syndrome (1). With the progression of NAFLD, its non-reversible form, non-alcoholic steatohepatitis (NASH), is involved in the development of cirrhosis and hepatocellular carcinoma (HCC), which lead to fatty liver with the presence of hepatocellular inflammatory injury and fibrosis (2, 3). In addition, NAFLD is associated with increased incidence of metabolic diseases, such as insulin resistance, type II diabetes, and hypertriglyceridemia. A few years ago, the American Association for the Study of Liver Disease guidelines suggested that only biopsy-proven NASH should be applied for medical treatment, but there are currently no other approved treatments for NAFLD except for losing weight through diet and exercise (4). But it is hard to achieve and sustain. There have been several drugs tested for the treatment of NAFLD, which including glitazones, vitamin E, and liraglutide, but they are not recommended due to their discordant results. Moreover, in 2017, there are no approved drug treatments for NAFLD and NASH (5). Therefore, finding an agent that can protect against hepatic steatosis may be a feasible strategy to treat NAFLD (6).

Adenosine monophosphate (AMP)-activated protein kinase (AMPK), a highly conserved energy transducer, provides energy for the cell by way of fatty acid metabolism, which is activated by AMP/ATP ratios and plays a crucial role in cardiovascular diseases, metabolic disorders, and inflammatory diseases (7). Moreover, AMPK also plays a key role in lipid metabolism in the liver and regulates the phosphorylation and inactivation of acetyl-CoA carboxylase (ACC), which results in inhibition of adipogenesis and prevention of fatty acid transport into the mitochondria for oxidation (8). In addition, it is well known that sterol regulatory element-binding protein 1c (SREBP-1c) is a crucial transcription factor that can adjust the synthesis of fatty acids and triglycerides (TGs) in the liver. Liver X receptor (LXR) induces SREBP-1c expression by binding to the LXR response element, but AMPK regulates LXR activity by phosphorylation, which contributes to the inhibition of SREBP-1c expression (9). AKT kinase (AKT) is a serine/threonine protein kinase that can be closely connected with AMPK (10). Despite this discovery, there appear to be different mechanisms by which AKT activity negatively regulates phosphorylation of AMP-activated protein kinase (11). Hence, understanding how these two kinases interact in this experiment may provide insights into NAFLD. Interestingly, peroxisome proliferator-activated receptors (PPARs) are ligand-inducible nuclear receptors that modulate the several biological processes disturbed in obesity, including inflammation, lipid and glucose metabolism, and overall energy homeostasis (12–14). Previous research has reported that mice lacking peroxisome proliferator-activated receptor α (PPARα) have an impaired response to fasting, characterized by fatty liver (15). In addition, some evidence demonstrates that PPARα agonists are also activated by AMP-activated protein kinase (16, 17).

Many pharmacology studies have demonstrated that natural compounds have protective effects not only against steatosis and inflammation but also, most of all, against fibrosis, which is the more important determinant of clinical outcomes in NAFLD (18, 19). Moreover, the natural compound resveratrol showed effects on metabolic health and aging by activating AMPK (20, 21). This natural compound possesses the ability to reduce liver fat accumulation by different mechanisms, including decreased lipogenesis by downregulation of SREBP-1 and ACC and increased fatty acid oxidation (22–24). Magnolol (MG), a kind of lignin, is extracted from *Magnolia officinalis* and has antifungal (25), anticancer (26), antioxidant, and hepatoprotective effects (27). Previous studies showed that MG could ameliorate fat accumulation, insulin resistance, and adipose inflammation (28, 29). Moreover, MG has been reported to play a critical role in regulating inflammatory responses and possesses strong anti-inflammatory effects. In a recent study, MG effectively suppressed lipopolysaccharide-induced inflammatory responses by inhibiting TLR4-mediated nuclear factor-kappa B (NF-κB) pathways in uterine epithelial cells (30) and activating the Nrf2/HO-1 signaling pathway in mouse macrophages (31). Liang et al. also found that MG could reduce TNF-α-induced vascular cell adhesion molecule-1 expression in endothelial cells (32). In addition, the literature reported that MG could regulate LXR, a nuclear receptor that is closely associated with lipid metabolism (33). If MG could inhibit inflammation and regulate the balance of energy metabolism in lipid accumulation progress, MG would ameliorate the development of NAFLD. In this paper, we show a new molecular mechanism that MG, as an agonist for PPARα, is closely associated with the activation of AMPK and AKT in the regulation of steatosis and hyperlipidemia. We also demonstrate that MG inhibits activation of NF-κB by blocking mitogen-activated protein kinase (MAPK) signal pathways in lipid accumulation course, which is not seen in other scientific researches.

## Materials and Methods

### Reagents and Chemical

Magnolol, purity ≥98%, was purchased from Chengdu Reference Products (Chengdu, China). Dimethylsulfoxide (DMSO), oleic acid (OA), tyloxapol (Ty), and Oil Red O were obtained from Sigma-Aldrich (St. Louis, MO, USA). An Oil Red O stain kit (for cultured cells) was purchased from Solarbio Science & Technology (Beijing, China). Reactive oxygen species (ROS), TG, and total cholesterol (TC) assay kits were provided by Jiancheng Bioengineering Institute of Nanjing (Nanjing, Jiangsu, China). Human TNF-α enzyme-linked immunosorbent assay (ELISA) kits were provided by BioLegend (CA, USA). U0126, SB203580, SP600125, and LY290042 (specific inhibitors of ERK1/2, P38, JNK1/2, and AKT, respectively) and antibodies against AMPKα, P-AMPKα, AMPKβ, P-AMPKβ, adenosine ACC, P-ACC, P-ERK1/2, ERK1/2, P-JNK1/2, JNK1/2, P-P38, P38, IκB, P-IκB, and P-P65 were purchased from Cell Signaling Technology (Boston, MA, USA). AKT, P-AKT (Thr 308) and GAPDH antibodies were purchased from Affinity (OH, USA). Antibodies against P65, SREBP-1c and β-actin were purchased from Proteintech (Boston, MA, USA). PPARα and compound c (inhibitor of AMPK) were purchased from Abcam (Cambridge, MA, USA). HRP-conjugated goat anti-rabbit and goat anti-mouse antibodies were provided by Boster (CA, USA). All other chemicals were of reagent grade.

### Animals

Male C57/BL6 mice (6–8 weeks), weighing approximately 18–22 g each, were purchased from Liaoning Changsheng Biotechnology (Liaoning, China). All animals were given adequate food and water *ad libitum*. The animals were housed in clean cages at 24 ± 1°C, with a 12 h light/dark cycle and relative humidity of approximately 40–80%. All studies were performed in accordance with the Guide for the Care and Use of Laboratory Animals published by the US National Institutes of Health. This study was reviewed and approved by the Animal Welfare and Research Ethics Committee at Jilin University.

### *In Vitro* Study

#### Cell Culture

The human HCC cell line HepG2 was obtained from the China Cell Line Bank (Beijing, China). HepG2 cells were cultured in high glucose DMEM containing 10% FBS and 0.5% penicillin–streptomycin in a humidified atmosphere containing 5% CO_2_ and 5% air at 37°C. Before the start of the extracorporeal experiment, cells were cultured with medium for 24 h.

#### MTT Assay

HepG2 cells were seeded at a density of 5 × 10^3^ cells/well in 96-well plates and incubated in a sterile incubator for 24 h. Then, the cell culture medium was discarded, and the cells were treated with different concentrations of MG (0–64 µg/ml) and OA (0–960 µM). After 24 h, 20 µL of MTT (5 mg/mL) was added to plates, and the cells were incubated for an extra 4 h. The supernatant was discarded, and DMSO (150 μL/well) was added to each well. The optical density was measured at 570 nm on a microplate reader (TECAN, Austria).

#### OA-Induced Steatosis

HepG2 cells were cultured in a 24-well plates (1 × 10^4^ cells/well), incubated for 24 h, and then given different concentrations of OA (30, 60, 120, 240, 480, and 960 µM) for another 24 h. The control group received medium containing BSA. The usable dosage was assessed by cell viability and lipid accumulation.

#### Oil Red O Staining for Cell Culture

Cells (1 × 10^4^/well) were treated with various OA concentrations (30, 60, 120, 240, and 480 µM) or incubated with an OA (120 µM) mixture with or without MG for 24 h. The culture medium was discarded, and cells were soaked with paraformaldehyde (4%) at room temperature for 30 min and washed with PBS three times. After 15 min of incubation with freshly prepared Oil Red O stain solution, slides were immersed in 60% isopropanol for 20–30 s. After swashing lightly in distilled water and PBS, the cells were treated with Mayer hematoxylin staining for 1–2 min, washed with ORO buffer for 1 min, and observed by light microscopy. The degree of Oil Red O staining was analyzed with Image-Pro plus 6.0. The mean optical density (MOD) was obtained as follows: MOD = IOD/SUM area.

#### Measurement of TG and TNF-α Levels

HepG2 cells were grown in 24-well plates (1 × 10^4^ cells/well) for 24 h and then pretreated with OA (0–480 µM) or a combination of OA (120 µM) and MG (4 µg/mL) for 24 h. The cell supernatant was collected to measure TNF-α level according to the ELISA manufacturer’s protocol. Then, the cells were exposed to Triton X-100 (2%) for 40 min. Finally, the intracellular TG levels were quantified by test kits (Nanjing, Jiangsu, China). The optical density was measured at 450 and 510 nm on a microplate reader (TECAN, Austria).

#### Measurement of ROS

HepG2 cells (5 × 10^3^ cells/well) were cultured in 96-well plates for 24 h and then pretreated with OA (120 µM) and MG (4 µg/mL) for another 24 h. The cells were grown with 10 µM of DCFH-DA for 30 min. Subsequently, the medium was removed, and the cells were dissociated with trypsin. Next, cells were collected to detect fluorescence. The optical density was measured at excitation and emission wavelengths of 485 and 525 nm, respectively, on a multidetection reader.

#### Western Blot

HepG2 cells (4 × 10^5^ cells/mL) were collected from 6-well plates to detect the protein expression. The cells were lysed for 30 min in radioimmunoprecipitation assay (RIPA) buffer containing 1 mM of phenylmethanesulfonyl fluoride. All protein concentrations were measured using the Pierce BCA protein assay kit (Thermo, USA). Then, 20 µg of sample proteins was transferred onto a polyvinylidene difluoride membrane following separation on a 12% SDS-polyacrylamide gel. Next, the membrane was blocked with 5% non-fat dry milk in TBST and shaken continuously on the table for 1 h at room temperature. Then, the membrane was washed once with TBST for 5 min. The membrane was soaked with primary antibody (1:1,000) overnight at 4°C and then washed with TBST three times for 5 min. Finally, the membrane was incubated with horseradish peroxidase-conjugated secondary antibodies (1:5,000) at room temperature for 1 h. After three washes, the bands were visualized by chemiluminescence (ECL) western blotting detection system, and the results were analyzed using ImageJ gel analysis software.

#### Immunofluorescence Analyses

HepG2 cells (5 × 10^3^ cells/well) were plated onto 96-well plates and fixed with 4% paraformaldehyde for 15 min then washed three times with PBS and incubated with 0.25% or 0.5% Triton X-100 for 10 min at room temperature to augment permeability. Cells were washed three times with PBS, incubated for 1 h with 2% BSA at room temperature, and then incubated at 4°C overnight with the primary antibody in PBS. The second day, after three PBS washes, cells were incubated for 1 h with the secondary antibody in a dark room and then washed three times with PBS. DAPI (1 µg/mL) was used for nuclear counterstaining for 10 min, and then cells were washed one time. The cells were imaged using a confocal laser scanning microscope (Olympus, Tokyo, Japan).

### *In Vivo* Study

#### Tyloxapol-Induced Hyperlipidemia in C57/BL6 Mice

In brief, the C57/BL6 mice were randomly divided into five groups: control (saline), Ty (500 mg/kg), MG (20 mg/kg), MG (10 mg/kg) + Ty (500 mg/kg), and MG (20 mg/kg) + Ty (500 mg/kg). The mice fasted for 12 h and were then intraperitoneally injected with MG (10 and 20 mg/kg). One hour later, mice were given intraperitoneal injection (i.p.) of Ty (500 mg/kg). Twelve hours later, the animals were sacrificed, after which the blood and liver tissues were collected for future research.

#### Histopathological Evaluation of the Liver Tissues

Liver tissues were fixed in normal 10% neutral-buffered formalin and dehydrated in different concentration of ethanol. They were then embedded in paraffin and sliced into 4-μm-thick sections. Subsequently, the sections were deparaffinized, rehydrated, and stained with hematoxylin and eosin to evaluate liver tissue pathology under a light microscope.

#### Oil Red O Staining for Animals

Fresh liver tissues acquired from the sacrificed mice were embedded in Tissue-Tek OCT compound (Sakura Finetek, Torrance, CA, USA) and frozen at −80°C. Tissue sections of 5 µm thickness were cut with a freezing microtome (Leica, Tokyo, Japan). The sections were mounted on slides and then immersed in a 10% formalin solution for 10 min. The slides were stained with Oil Red O solution for 15 min, rinsed with 60% isopropanol, and then slightly stained with Mayer’s hematoxylin solution for 2–3 min. Next, the slides were washed with running water. After that, the liver tissues were photographed and observed under a light microscope.

#### Measurement of TG and TC

To determine lipid accumulation, mice were given intraperitoneal injections of Ty (500 mg/kg) at 3, 6, or 12 h. The blood was collected for lipid analysis. Next, mice were pretreated with a range of different concentrations of MG for 1 h. The mice were then treated with Ty for 12 h. Subsequently, serum levels of TG and TC were analyzed using the corresponding detection kits in accordance with the manufacturer’s instructions (Jiancheng, China).

#### Western Blot

An approximately 100 mg sample of each liver tissue was homogenized and dissolved in RIPA lysis buffer to analyze protein expression. The sample was subjected to the next procedure as described in Section “Western Blot.”

### Statistical Analysis

All data were analyzed by using GraphPad Prism 6.0 software and in accordance with normal distribution and homogeneity analysis. All experiments were carried out in duplicate or triplicate with at least three biological replicates. The results are described as mean ± SD from three independent experiments. Differences between the means of groups were statistically analyzed by ANOVA followed by Tukey’s test using 95% confidence intervals as the significance standard (*p* < 0.05 and *p* < 0.01).

## Results

### OA-Induced Lipid, TG Accumulation, and Cytotoxicity in HepG2 Cells

**Figure 1:**
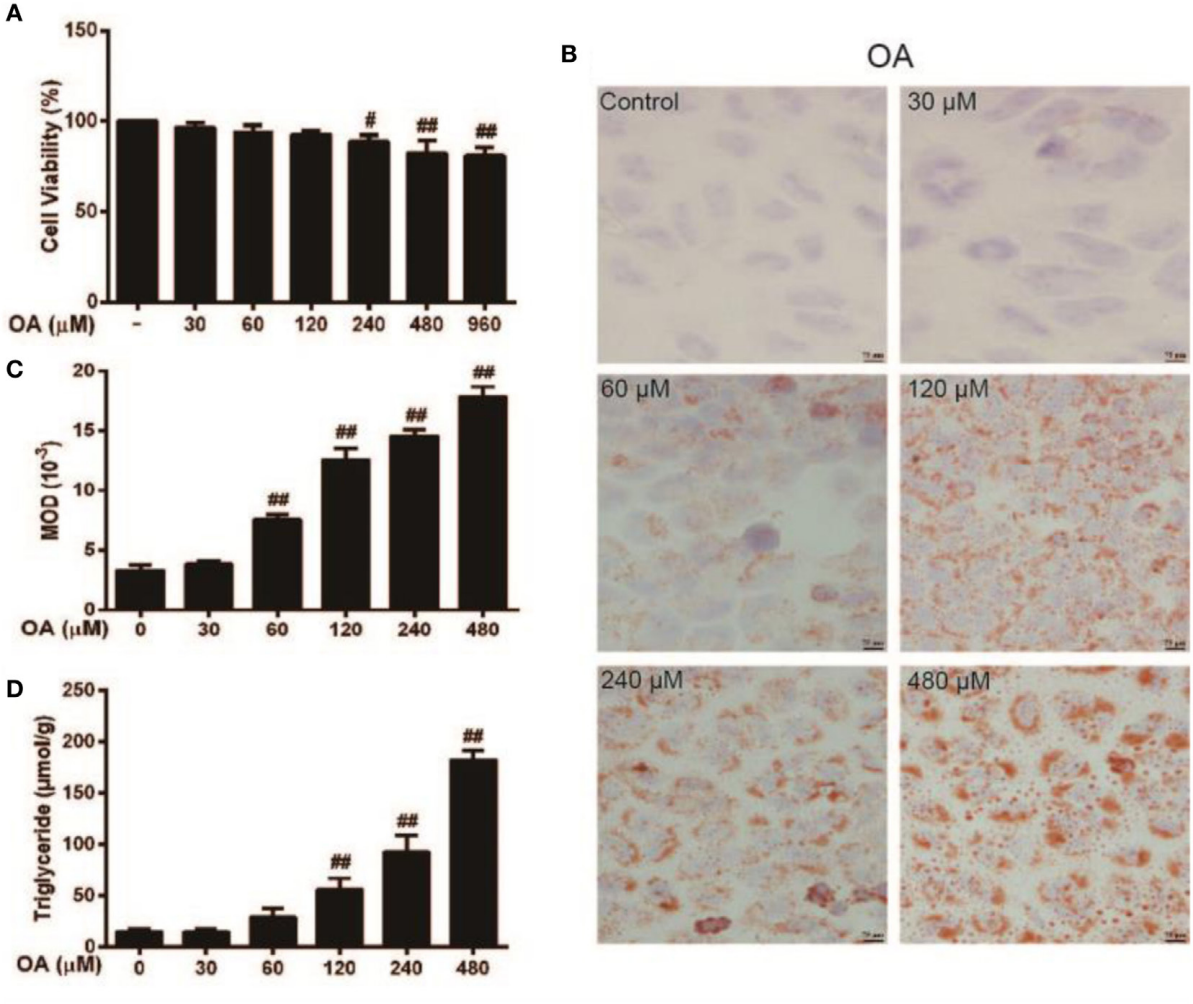
Effect of oleic acid (OA)-induced triglyceride (TG) accumulation and cytotoxicity in HepG2 cells. **(A)** HepG2 cells were exposed to various concentrations of OA (0, 30, 60, 120, 240, 480, and 960 µM) for 24 h, and cell viability was subsequently determined by MTT assay. **(B,C)** The effect of OA (30, 60, 120, 240, and 480 µM) on TG accumulation in HepG2 cells. Original magnification was 200×, and scale bars represent 75 µm. **(D)** Effect of TG concentration on OA-induced HepG2 cells. All data are expressed as the mean ± SD of three independent experiments. Statistically significant at ^#^*p* < 0.05 and ^##^*p* < 0.01 vs. the control group (one-way ANOVA followed by Tukey’s analysis).

To determine the stability and cell viability of OA-induced steatosis *in vitro*, HepG2 cells were given 0–960 µM doses of OA for 24 h to stimulate steatosis. As is presented in Figure 1A, the optimal OA concentration required to induce a cytotoxic effect was 0–60 µM. However, according to Figures 1B–D, these results revealed that the levels of TG and Oil Red O staining were not enough to cause steatosis within the 0–60 µM range. In addition, the cell viability of cells treated with OA (120 µM) exceeded 90% in comparison with the control. OA (120 µM) was selected to evaluate steatosis. In comparison with the control (HepG2 cells without OA), OA (120 µM) caused TG content and lipid accumulation (as determined by MOD) to noticeably increase. Thus, OA (120 µM) was selected as the optimal concentration to induce steatosis.

### MG Suppressed OA-Induced TG Accumulation in HepG2 Cells

**Figure 2:**
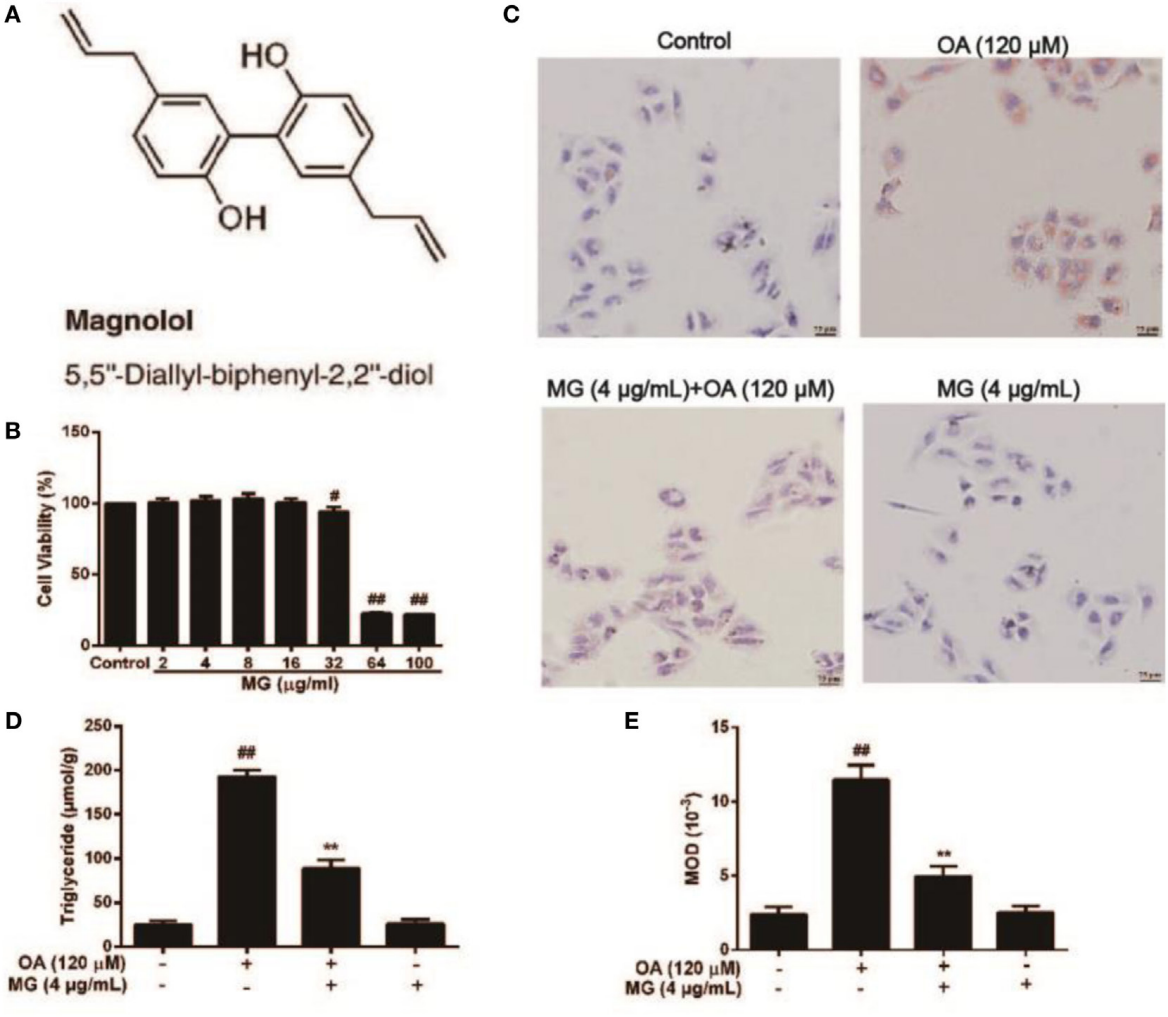
Effect of magnolol (MG) on oleic acid (OA)-induced triglyceride (TG) accumulation in HepG2 cells. **(A)** The chemical structure of MG. **(B)** Cytotoxicity of MG in HepG2 cells. HepG2 cells were treated with increasing concentrations of MG (2, 4, 8, 16, 32, 64, and 100 µg/mL) for 24 h, and then cell viability was measured by MTT assay. **(C,E)** Oil Red O staining. HepG2 cells were incubated with MG (4 µg/mL) and OA (120 µM) for 24 h. **(D)** The inhibitory effect of MG on OA-induced TG accumulation. All data are expressed as the mean ± SD of three independent experiments. Statistically significant at ^#^*p* < 0.05 and ^##^*p* < 0.01 vs. the control group and **p < 0.01 vs. the OA-treated group (one-way ANOVA followed by Tukey’s analysis).

The chemical structure of MG is described in Figure 2A (34). To evaluate the cytotoxicity of MG, HepG2 cells were treated with MG (0–100 µg/mL), and cell viability was determined by MTT assay (Figure 2B). Our results demonstrated that MG (0–16 µg/mL) did not alter cell viability during 24 h of exposure. To avoid experiment errors and verify the efficiency of MG, we selected a safe and effective dose of MG (4 µg/mL) to use in future studies. Afterward, TG and MOD were measured by the TG test kit and Oil Red O staining, respectively. As is described in Figures 2C–E, OA induced significant activation of TG and lipid accumulation, but MG (4 µg/mL) attenuated this effect.

### MG Regulated the Expression of AMPKα, AMPKβ, ACC, and AKT Protein in HepG2 Cells

**Figure 3:**
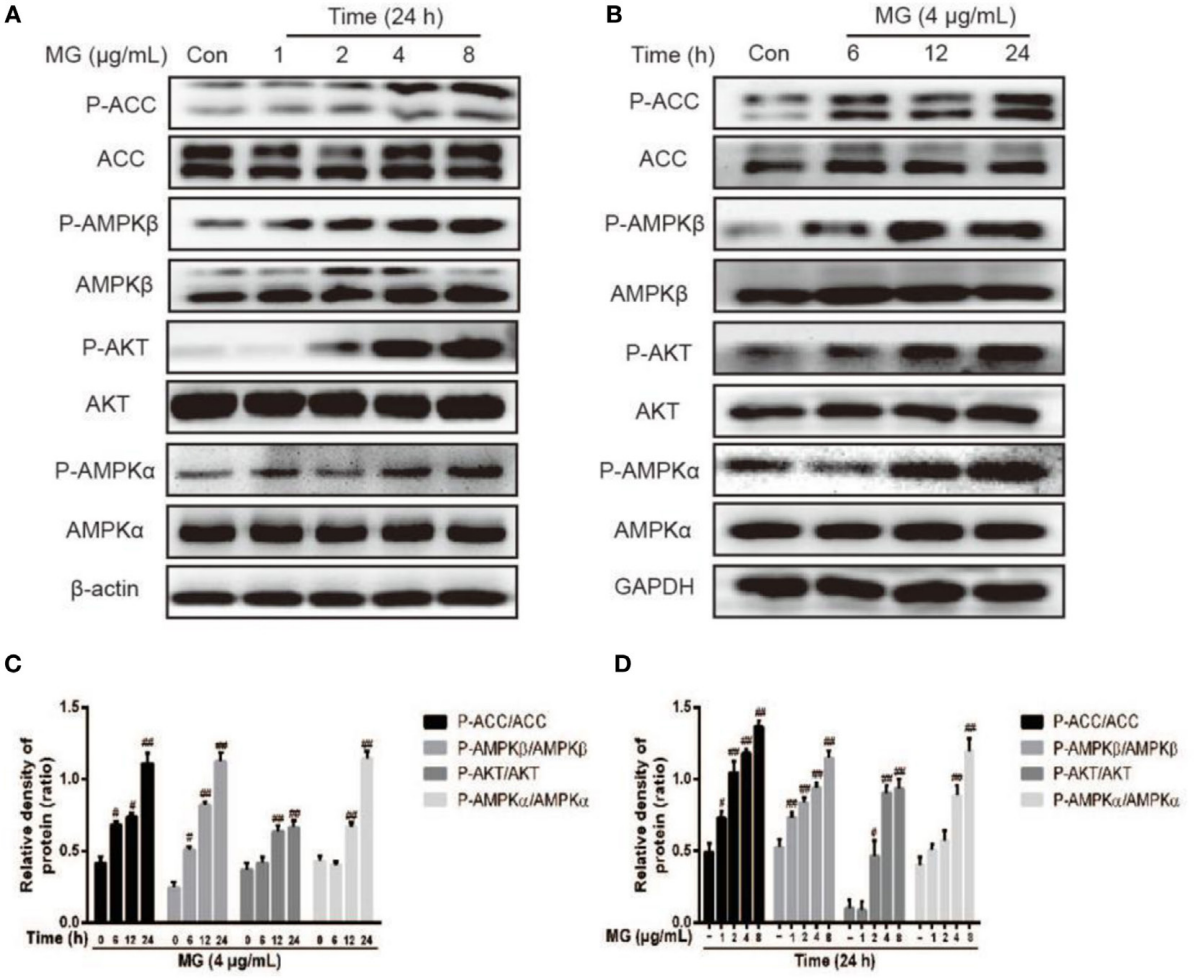
Effect of magnolol (MG) on the activation of AMPKα, AMPKβ, acetyl-CoA carboxylase (ACC), and AKT in HepG2 cells. **(A)** HepG2 cells were treated with increasing concentrations of MG (1, 2, 4, and 8 µg/mL) for 24 h. **(B)** Cells were cultured with MG (4 µg/mL) for three time points (6, 12, or 24 h). All cell lysates were prepared and detected by western blot analysis for phosphorylated and total AKT, AMPKα, AMPKβ, and ACC protein expression. **(C,D)** Quantification of P-AKT/AKT, P-AMPKα/AMPKa, P-AMPKβ/AMPKβ, and P-ACC/ACC protein expression was performed by software analysis. All data are expressed as the mean ± SD of three independent experiments. Statistically significant at ^#^*p* < 0.05 and ^##^*p* < 0.01 vs. the control group (one-way ANOVA followed by Tukey’s analysis).

HepG2 cells were treated with MG (4 µg/mL) at three time points (6, 12, or 24 h) or MG (1, 2, 4, and 8 µg/mL) at 24 h, and the total protein was isolated from these cells for further analysis. As is shown in Figures 3A–D, MG (4 µg/mL) clearly increased phosphorylation of AMPKα, AMPKβ, ACC, and AKT at 24 h. These observations indicated that cells treated with 4 µg/mL of MG for 24 h apparently upregulated the expression of antihyperlipidemia-related protein in HepG2 cells.

### MG Ameliorated OA-Induced Hepatic Steatosis by Regulating AKT, AMPK, SREBP-1c, and PPARα in HepG2 Cells

**Figure 4:**
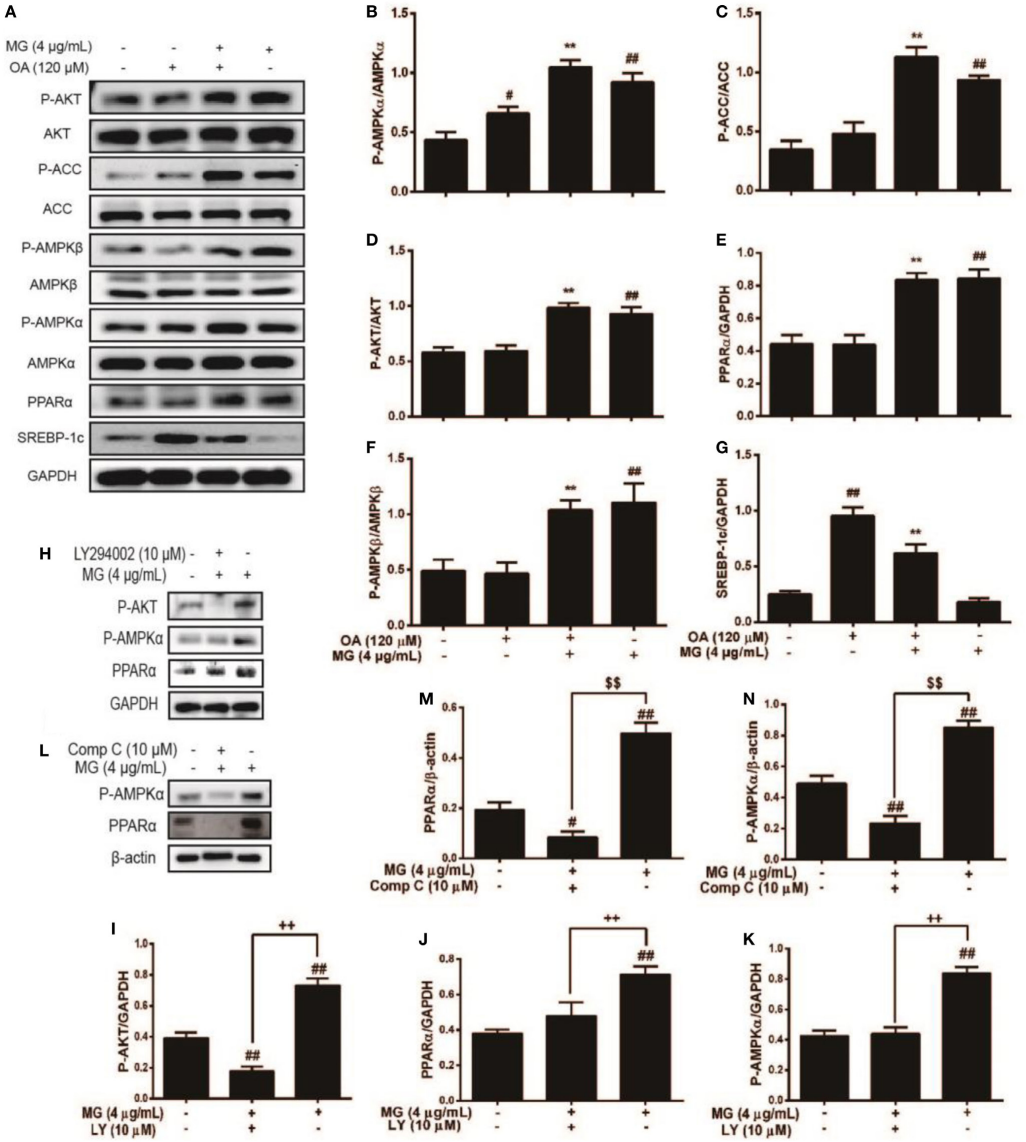
Effect of magnolol (MG) regulation of multiple signaling pathways on oleic acid (OA)-induced hepatic steatosis in HepG2 cells. Cells were stimulated by MG (4 µg/mL) with or without OA (120 µM) for 24 h. **(A)** Protein expression [including P-AKT, AKT, P-acetyl-CoA carboxylase (ACC), ACC, P-AMPKα, AMPKα, P-AMPKβ, AMPKβ, peroxisome proliferator-activated receptor α (PPARα), and sterol regulatory element-binding protein 1c (SREBP-1c)] was analyzed by western blot. **(H,L)** Cells were pretreated with or without LY294002 (inhibitor of AKT) and compound c (inhibitor of AMPK) for 1 h, followed by incubation with MG (4 µg/mL) for 24 h. **(B–G,I–K,M,N)** Relative expression levels of all proteins were quantified by density analysis. Analyzed proteins include phosphorylated and total AKT, AMPKα, AMPKβ, and ACC. In addition, total PPARα and SREBP-1c were also detected. Fold induction results were acquired from three independent experiments and are presented as the mean ± SD. Statistically significant at ^##^*p* < 0.01 vs. the control group, *p < 0.05 and **p < 0.01 vs. the OA-treated group, ^$$^*p* < 0.01 vs. the compound c-treated group, and ^++^*p* < 0.01 vs. the LY294002-treated group (one-way ANOVA followed by Tukey’s analysis).

**Figure 5:**
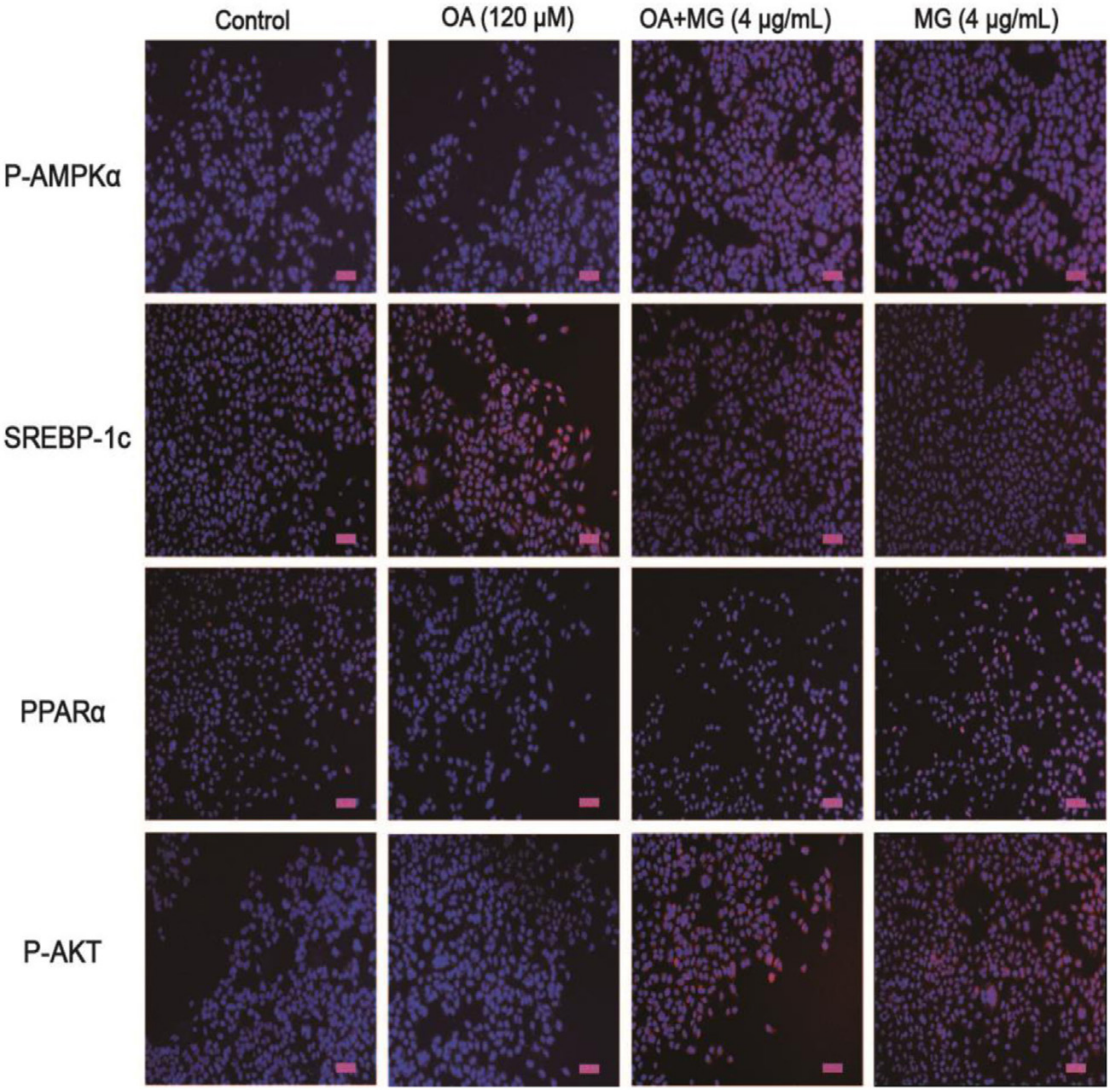
Effect of magnolol (MG) on the expression of proteins related to hepatic steatosis in HepG2 cells. Cells were exposed to MG (4 µg/mL) with or without oleic acid (OA) (120 µM) for up to 24 h. Afterward, immunofluorescence for peroxisome proliferator-activated receptor α (PPARα), SREBP-1c, P-AKT, and P-AMPKα (red) and the nuclear dye DAPI (blue), and scale bars represent 100 µm. All data are expressed as the mean ± SD of three independent experiments.

Peroxisome proliferator-activated receptor α is an important factor in hyperlipidemia. Thus, we investigated whether MG pretreatment could attenuate OA-triggered lipid accumulation. As is shown in Figures 4A,E, OA did not induce PPARα activation, but MG clearly augmented PPARα protein expression. Next, we analyzed the upstream protein SREBP-1c, which is associated with lipogenesis and is a sign of lipid abnormality (Figures 4A,G). OA visibly induced high levels of SREBP-1c protein expression, but MG prevented this accumulation. Various signal transduction pathways, including the AKT, AMPK, and ACC pathways, are strongly related to the regulation of SREBP-1c expression. To further investigate whether MG-inhibited SREBP-1c activation is regulated by these signaling pathways, the effect of MG on the AKT, AMPK, and ACC pathways was explored. As is described in Figures 4A–D,F, the results showed that MG clearly activated P-AMPKα, P-AMPKβ, P-AKT, and P-ACC protein expression in OA-induced hepatic steatosis. Next, we wanted to confirm that MG increased PPARα protein expression by activating the AMPK and AKT pathways. Cells were treated with inhibitors of AKT (LY294005) and AMPK (compound c), and the results demonstrated that these inhibitors also blocked PPARα protein expression (Figures 4H–N). These investigations indicated that MG regulated PPARα translocation *via* the activation of AMPK and AKT signaling in HepG2 cells. Moreover, the immunofluorescence results also demonstrated that MG reliably activated the AMPK, AKT, and PPARα pathways and inhibited the SREBP-1c pathway in OA-induced hepatic steatosis (Figure 5).

### MG Suppressed OA-Activated NF-κB Protein Expression in HepG2 Cells

**Figure 6:**
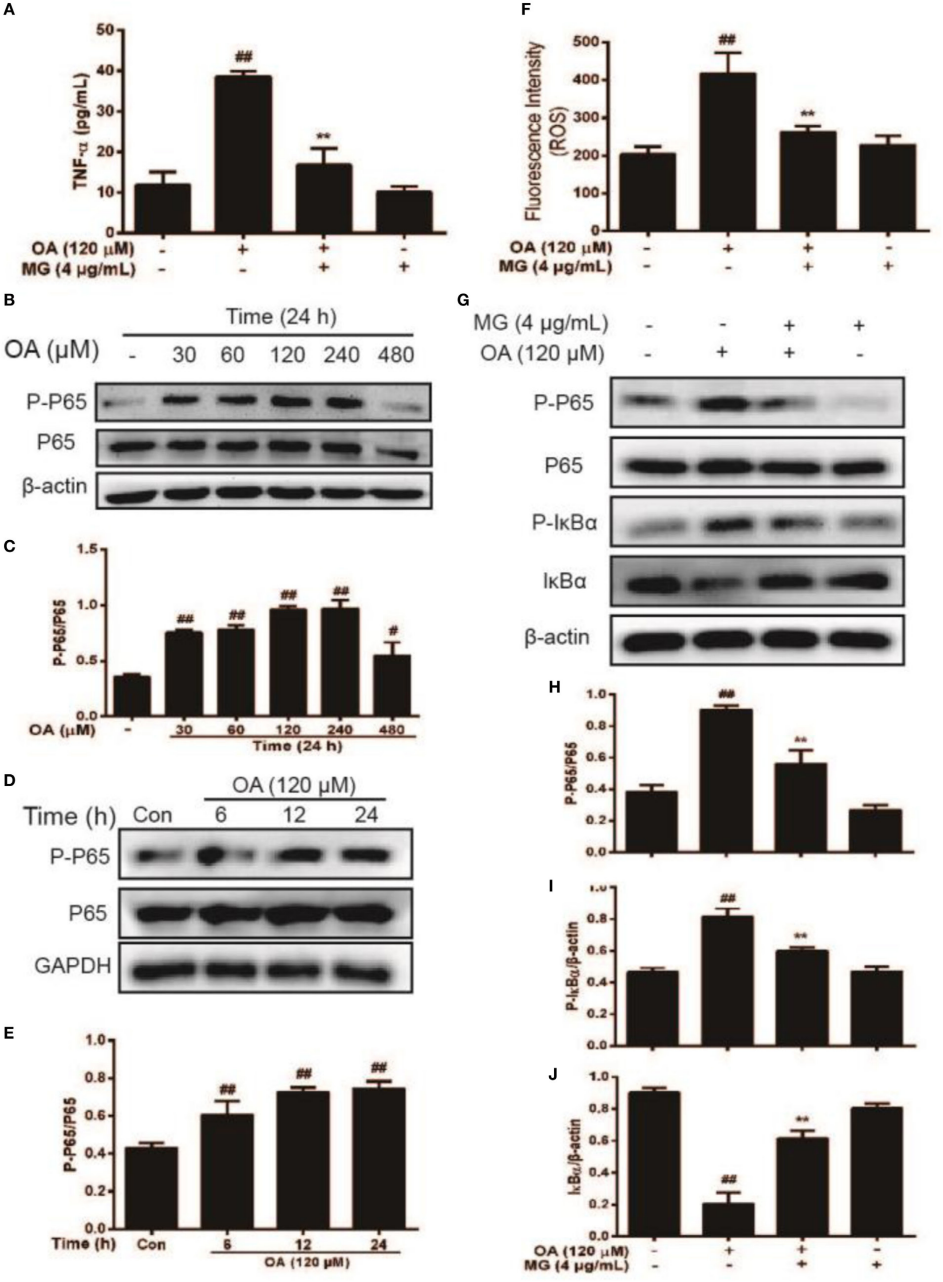
Effect of magnolol (MG) on oleic acid (OA)-activation of nuclear factor-kappa B protein expression in HepG2 cells. Cells were exposed to MG (4 µg/mL) with or without OA (120 µM) for up to 24 h. Next, **(A)** TNF-α production was determined by enzyme-linked immunosorbent assay at 450 nm, and **(F)** reactive oxygen species release was determined by chemiluminescence assay at excitation and emission wavelengths of 485 and 535 nm, respectively. **(B,C)** HepG2 cells were cultured with OA (30, 60, 120, 240, and 480 µM) for 24 h. P65 and P-P65 protein expression was detected by western blot. **(D,E)** Cells were exposed to OA (120 µM) for 6, 12, or 24 h. Protein expression of P-P65 and P65 was measured by western blot. **(G–J)** At the indicated doses, cells were treated with MG (4 µg/mL) in the presence or absence of OA (120 µM) for 24 h. Protein expression of P-P65, P65, P-IκBα, and IκBα was analyzed by western blot. Quantification of the relative expression of P-P65/P65, P-IκBα/β-actin, and IκBα/β-actin was obtained by density analysis. All data are expressed as the mean ± SD of three independent experiments. Statistically significant at ^#^*p* < 0.05 and ^##^*p* < 0.01 vs. the control group and **p < 0.01 vs. the OA-treated group (one-way ANOVA followed by Tukey’s analysis).

Both ROS and TNF-α inflammatory factor accumulation are closely connected with the activation of NF-κB (Figures 6A,F). Thus, we detected the OA-induced activation of phospho-p65, and the preventive effect of MG on the OA-induced NF-κB elevation. HepG2 cells were cultured with increasing concentrations of OA (0–480 µM) for 24 h to stimulate the cells (Figures 6B,C). Our results indicated that pretreatment of OA activated phospho-P65 protein expression in a dose-dependent manner. Next, we chose an optimal concentration of OA (120 µM) and analyzed its effects at different time points to verify the time-dependent effects (Figures 6D,E). The optimum concentration and time point were found. Then, cells were treated with the drug to investigate the effect of MG. As is presented in Figures 6G–J, MG (4 µg/mL) effectively inhibited the OA-induced phospho-P65 and phospho-IκB protein expression.

### MG Suppressed OA-Activated MAPK Protein Expression in HepG2 Cells

**Figure 7:**
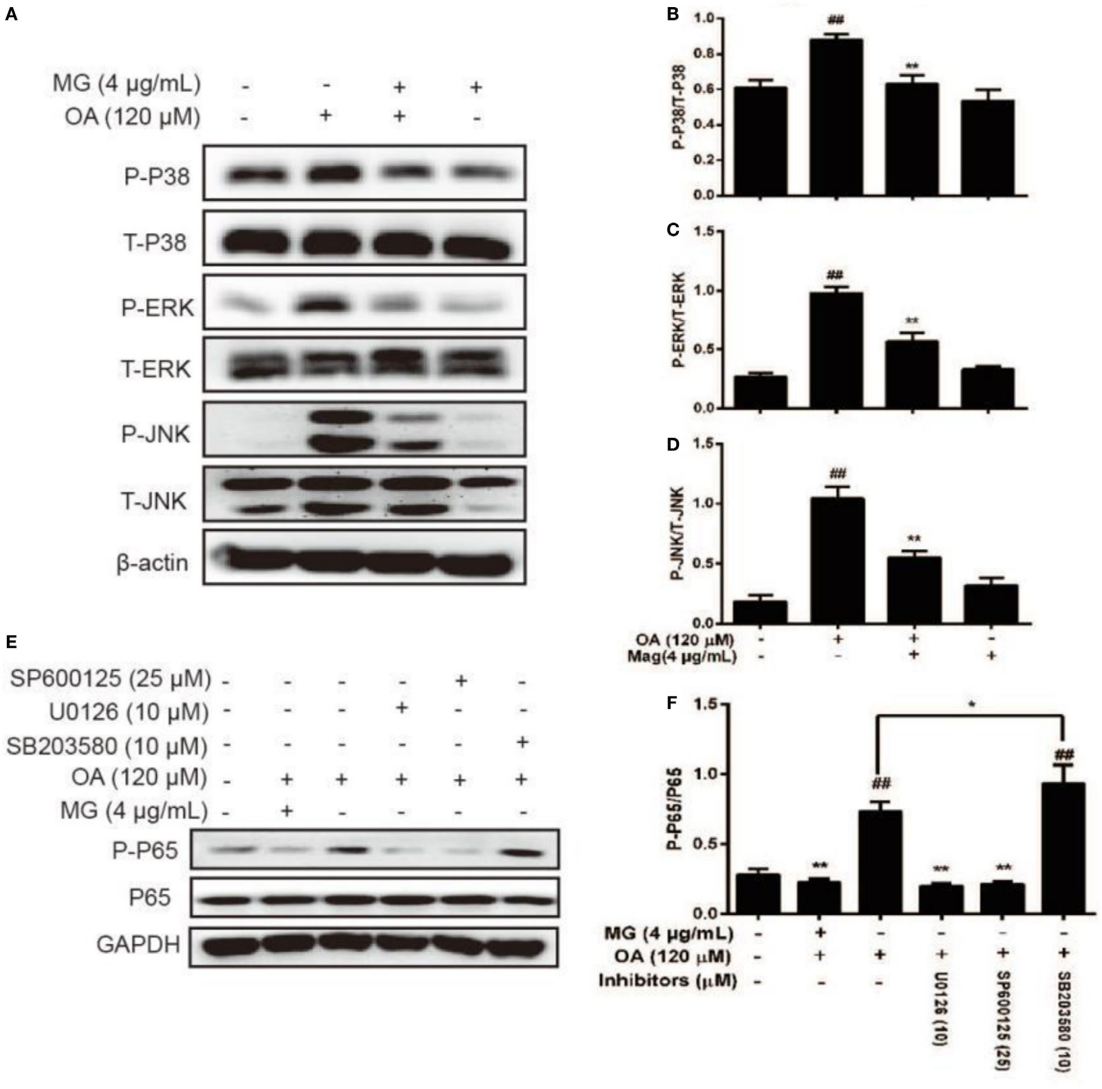
Effect of magnolol (MG) on oleic acid (OA)-activation of mitogen-activated protein kinase protein expression in HepG2 cells. Cells were incubated with MG (4 µg/mL) in the presence or absence of OA (120 µM) for 24 h. **(A)** Protein expression levels of P-P38, P38, P-c-Jun N-terminal kinase (JNK), JNK, P-extracellular regulated protein kinase (ERK), and ERK were measured by western blot analysis. **(B–D)** Quantification of the relative protein expression of P-P38/P38, P-JNK/JNK, and P-ERK/ERK was determined by photodensitometry. **(E)** SP600125, U0126, and SB203580 (inhibitors of JNK, ERK, and P38, respectively) were added to plates of cultured cells at 30 min or 1 h for protein inhibition and then mixed with OA (120 µM) for an additional 24 h. Protein expression levels of P-P65 and P65 were checked by western blot. **(F)** Quantification of the relative expression of P-P65/P65 was revealed by density analysis. The fold induction results were acquired from three independent experiments and presented as the mean ± SD. Statistically significant at ^##^*p* < 0.01 vs. the control group and *p < 0.05 and **p < 0.01 vs. the OA-treated group (one-way ANOVA followed by Tukey’s analysis).

Mitogen-activated protein kinase signaling pathways play an important role in the inflammatory process and are activated by the release of pro-inflammatory cytokines. The next step was to investigate whether MG inhibited NF-κB expression by attenuating MAPK signaling pathways. As is shown in Figures 7A–D, the pretreatment of cells with OA distinctly enhanced AMPK phosphorylation. Moreover, MG reversed this process. It is well known that MAPK family members include JNK1/2, P38, and ERK1/2. Furthermore, our results demonstrated that MG (4 µg/mL) ameliorated OA-induced phosphorylation of JNK1/2, ERK1/2, and P38. However, to further verify this effect, we used SP600125, U0126, and SB203580, which are the specific inhibitors of c-Jun N-terminal kinase (JNK), extracellular regulated protein kinase (ERK), and P38, respectively. The ERK and JNK inhibitors clearly inhibited OA-induced NF-κB activation (Figures 7E,F), but the P38 inhibitor did not suppress OA-induced NF-κB activation. These results indicated that MG mitigated OA-induced NF-κB activation by inhibiting ERK and JNK rather than by inhibiting P38.

### MG Treatment Alleviated Histopathological Changes and Lipid Accumulation in Tyloxapol-Induced Hyperlipidemia Mice

**Figure 8:**
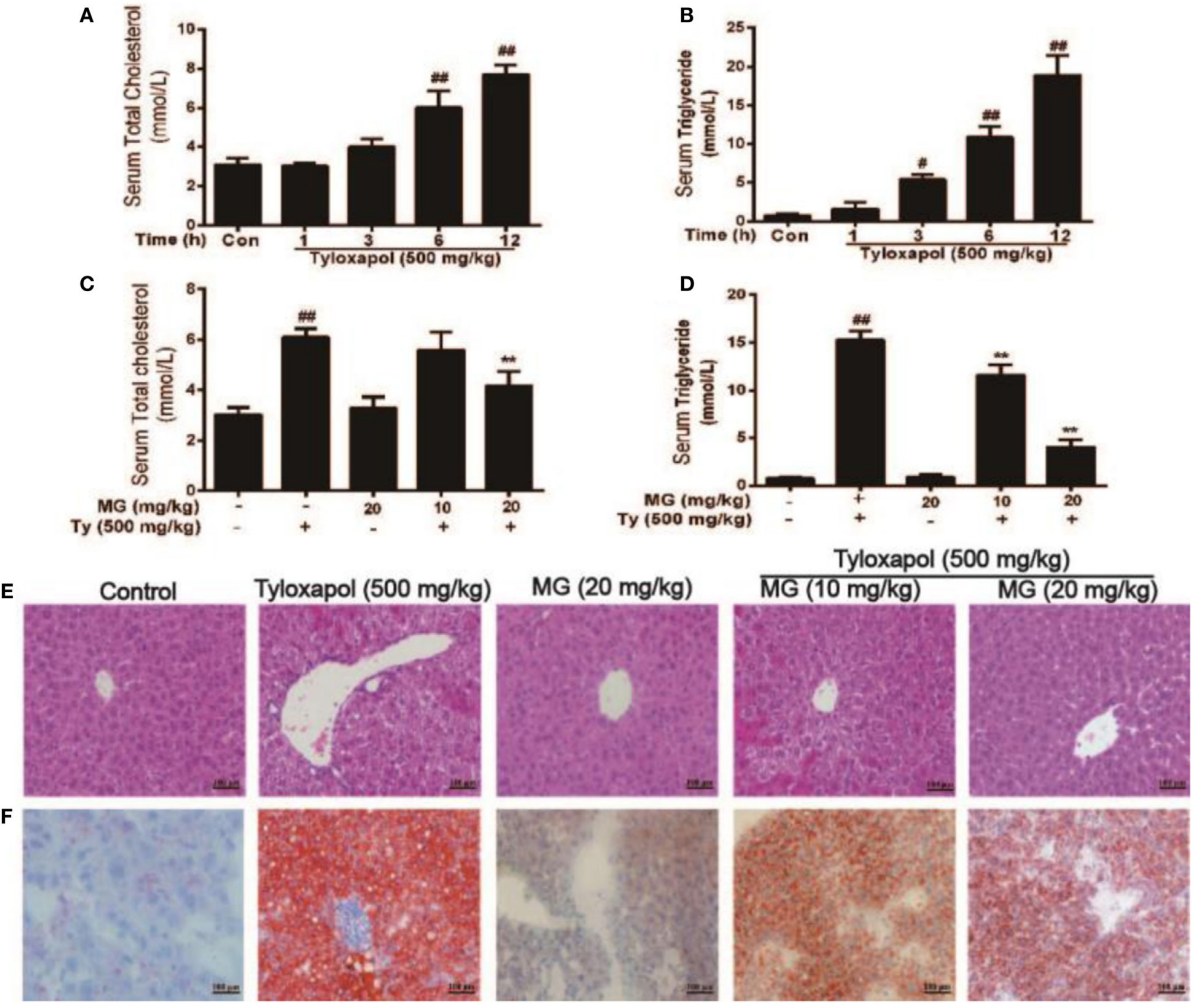
Effect of magnolol (MG) treatment on histopathological changes and lipid accumulation responses in tyloxapol (Ty)-induced hyperlipidemia mice. **(A,B)** Tyloxapol (500 mg/kg) was intraperitoneally injected into mice at four time points (1, 3, 6, or 12 h). Next, the serum was extracted to evaluate total cholesterol (TC) and triglyceride (TG) levels in mice. **(C,D)** At the indicted time points, mice were administered MG (10 and 20 mg/kg) for 1 h. Afterward, the mice were injected with Ty (500 mg/kg) for 12 h. Serum TC and TG were assayed using assay kits. **(E)** Hematoxylin and eosin staining of liver tissues (magnification 200×, scale bar = 100 µm). **(F)** Oil Red O staining of live tissues (magnification 200×, scale bar = 100 µm). **(E,F)** Representative histological images derived from mice of different groups were tested for lipid accumulation. Analysis of the sections suggested that Ty led to accumulation of hepatocyte vacuoles and lipid droplets. Interestingly, MG attenuated this situation. The amount of lipid droplets inside and between hepatocytes decreased. All data are expressed as the mean ± SD of three independent experiments. Statistically significant at ^#^*p* < 0.05 and ^##^*p* < 0.01 vs. the control group and **p < 0.01 vs. the Ty-stimulated group (one-way ANOVA followed by Tukey’s analysis).

As is illustrated in Figures 8A,B, we assessed the *in vivo* effects of MG in Ty-induced lipid accumulation and saw that Ty (500 mg/kg) dramatically increased the serum TG and TC levels at four time points (1, 3, 6, and 12 h). According to these results, Ty (500 mg/kg) was selected to stimulate for 12 h. Then, MG (10 and 20 mg/kg) reduced these increases (Figures 8C,D). Next, we investigated whether Ty (500 mg/kg) could induce hepatic steatosis at 12 h in mice. Histological examination of liver tissues showed that Ty treatment gives rise to partial hepatocellular vacuoles (Figure 8E). In addition, Oil Red O staining exhibited that Ty elevated hepatic TG in mice (Figure 8F). Furthermore, MG treatment ameliorated the elevation of hepatic TG and vacuole formation.

### MG Treatment Regulated the Protein Expression of AMPK, PPARα, AKT, and SREBP-1c in Tyloxapol-Induced Hyperlipidemia Mice

**Figure 9:**
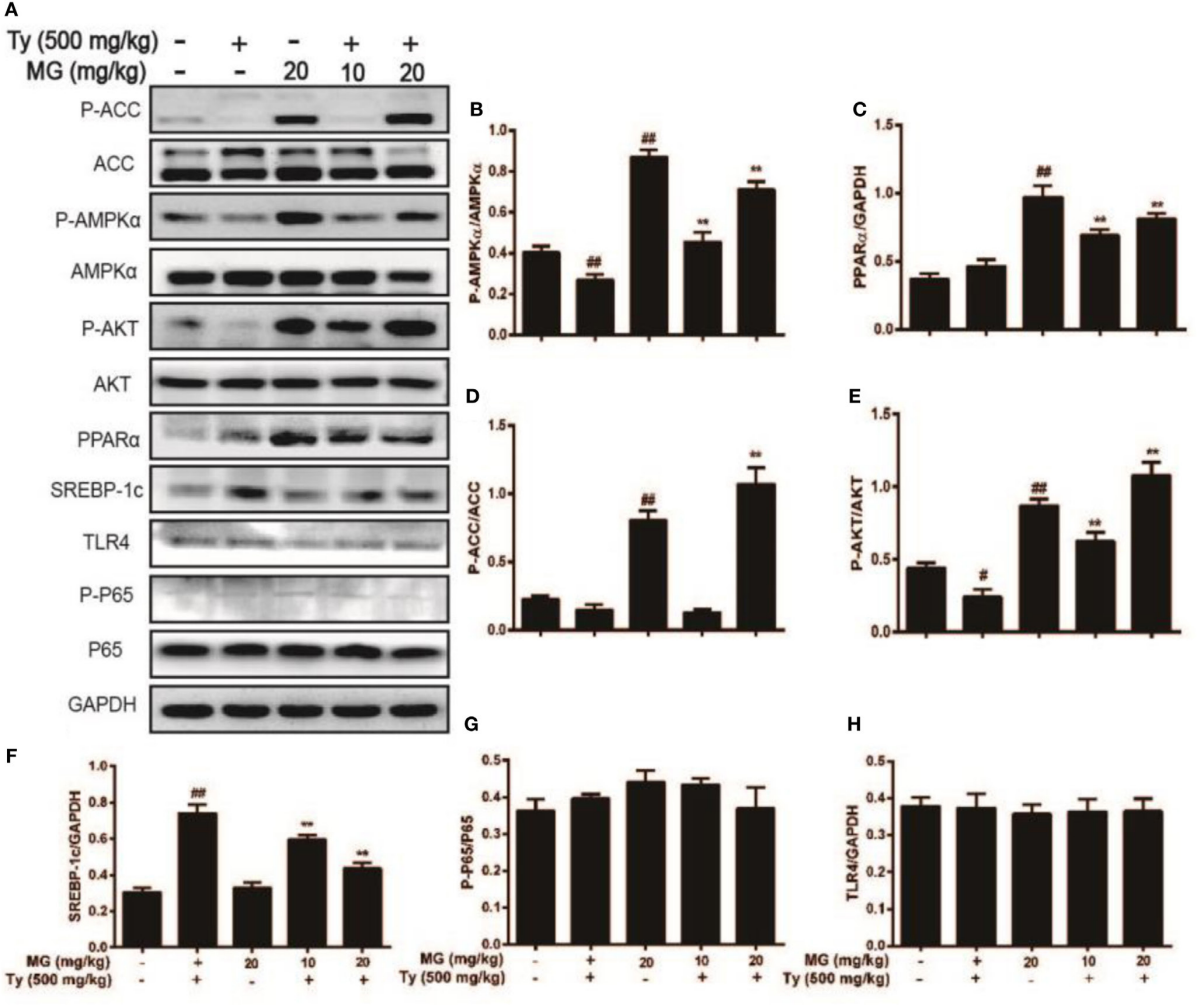
Effect of magnolol (MG) treatment on AMPK, peroxisome proliferator-activated receptor α (PPARα), AKT, and sterol regulatory element-binding protein 1c (SREBP-1c) in tyloxapol (Ty)-induced hyperlipidemia mice. Mice were administered MG (10 and 20 mg/kg) for 1 h. Afterward, the mice were injected with Ty (500 mg/kg) for 12 h. Liver tissues were gathered and analyzed by western blot. **(A)** Effects of MG on P-acetyl-CoA carboxylase (ACC), ACC, P-AMPKα, AMPKα, P-AKT, AKT, PPARα, SREBP-1c, P-P65, P65, and TLR4 were measured by western blot analysis. **(B–H)** Quantification of relative protein expression was performed by software analysis. All data are expressed as the mean ± SD of three independent experiments. Statistically significant at ^#^*p* < 0.05 and ^##^*p* < 0.01 vs. the control group and **p < 0.01 vs. the Ty-stimulated group (one-way ANOVA followed by Tukey’s analysis).

We showed that MG exposure ameliorated Ty-induced hepatic steatosis in live tissue. Next, we explored the molecular mechanism of MG. As is illustrated in Figures 9A–F, MG increased the protein expression of the antihyperlipidemia-associated proteins P-AMPKα, P-ACC, P-AKT, and PPARα and inhibited Ty-induced SREBP-1c protein levels in a dose-dependent manner. However, we did not find that Ty could activate the expression of the inflammatory proteins TLR4 and NF-κB at 12 h (Figures 9A,G,H).

## Discussion

Non-alcoholic fatty liver disease often gives rise to hepatic steatosis, which is mostly associated with abnormal lipid metabolism and lipid accumulation (6). AMPK has previously been shown to adjust hepatic fatty acid oxidation, inhibit cholesterol synthesis and TG synthesis, and repress adipocyte lipolysis and lipogenesis (35). Furthermore, one of the key pathways involved in AMPK regulation is the phosphorylation and inactivation of ACC (36). This enzyme converts acetyl-CoA to malonyl-CoA, which can inhibit carnitine palmitoyltransferase 1 and lead to fatty acid oxidation (37). In addition, protein kinase B (PKB), also known as AKT, is upstream of AMPK and associated with activation of AMPK protein expression (38). These findings indicate that the phosphorylation of AMPK involves AKT and ACC. Admixtures of MG and honokiol, pharmacological biphenolic compounds of *M. officinalis*, have been reported to regulate AMPK to repress steatosis (28). Therefore, MG may be the crucial element, but this conjecture has not been corroborated. Therefore, this study aimed to investigate whether MG modulates the phosphorylation of AMPK and alleviates steatosis and hyperlipidemia.

Subsequently, to dissect the step at which MG acts to regulate the anti-steatosis signaling pathway, we screened different concentrations and time points of MG treatment for their regulation of the signaling pathway. MG indeed activated the phosphorylation of AKT, AMPK, and ACC in HepG2 cells. OA is a type of fatty acid, which is used to model NAFLD in HepG2 cells and can clearly cause steatosis (39). MG mitigated TG content and lipid accumulation and noticeably elevated the phosphorylation of AMPK, ACC, and AKT in OA-induced hepatic steatosis in HepG2 cells. This indicated that MG might play a role in lipid metabolism. According to these cellular experiments, the mechanism of MG attenuation of OA-induced lipid accumulation was discussed but remained unconvincing. Thus, we continued to explore the effects of MG in mice. Tyloxapol is a kind of non-ionic surfactant, which blocks plasma lipolytic activity and induces TG-rich lipoproteins in mice (40). This mechanism is used to generate an acute mode of experimental hyperlipidemia, which is associated with NAFLD (41). In our studies, pretreatment with Ty significantly increased serum TG and TC content at 12 h in C57/BL6 mice. Liver histopathology results also verified this finding. Our results suggested that MG pretreatment inhibited the levels of serum TG and TC and ameliorated hepatic lipid accumulation. MG also clearly promoted liver AMPK, AKT, and ACC phosphorylation. During this process, we unexpectedly discovered that MG treatment promoted the expression of PPARα in OA-induced hepatic steatosis and Ty-stimulated hyperlipidemia. PPARα is the key regulatory transcription factor for lipid metabolism, and exposure to MG may contribute to the observed positive effects (42). Moreover, previous studies reported that both PPARα and AMPK could inhibit SREBP-1c generation, a liver lipogenic gene, which plays a key role in lipogenesis (42, 43). MG inhibited the increase in SREBP-1c in these models. However, in cell experiments in which MG was mixed with the inhibitors of AMPK and AKT, the trend toward increased PPARα levels was lost. These observations suggested that MG represses steatosis and hyperlipidemia *via* activating PPARα pathways, dependent on AKT/AMPK and suppresses SREBP-1c elevation.

Non-alcoholic fatty liver disease is a progressive disease that can develop into NASH through varied mechanisms and result in steatosis accompanied by inflammation and fibrosis (44). P65 (RELA) plays the essential role of transcription factor and is one member of the NF-κB family (45). Mutations in P65 are responsible for inflammatory bowel disease and defective immune response (46). As is already known, the RELA/P50 complex is mainly sequestered by IκBα in the cytosol. TNF-α activates the phosphorylation of IκBα, inducing rapid degradation of IκBα and the subsequent release of the P65/P50 complex. The P65 nuclear localization signal, which used to be sequestered by IκBα, is now exposed by this process, resulting in the rapid translocation of NF-κB (47). Previous research reported that the NF-κB pathway may be a possible target for assuagement of NASH (48). Therefore, P65 was detected by western blot in OA-induced HepG2 cells. These results verified that OA could enhance the phosphorylation of P65 in a dose-dependent and time-dependent manner in HepG2 cells. Then, we selected the effective OA concentration and treatment time point to explore the protective effects of MG. Not only did MG mitigate the phosphorylation of P65 and IκB but it also inhibited the release of TNF-α and ROS in OA-induced HepG2 cells. Furthermore, NF-κB activation is associated with the regulation of multiple cellular signaling pathways, including MAPK (49). We found that MG inhibited the phosphorylation of JNK, ERK, and P38 in OA-induced HepG2 cells, all of which are members of the MAPK signaling pathway. At the same time, pretreatment with inhibitors of JNK and ERK attenuated NF-κB activation, while inhibition of P38 did not have this effect. These data demonstrate that MG decreases TNF-α generation and phosphorylation of JNK and ERK to block the phosphorylation of NF-κB and IκB in the OA-induced inflammatory response in HepG2 cells. Pretreatment with Ty did not stimulate inflammation as demonstrated by detection of TLR4 and NF-κB expression for 12 h. We speculated that this conflict resulted from the NAFLD developmental processes in mice, as NASH is the most extreme form of NAFLD that can induce an inflammatory response (50). On the one hand, the mice treated with Ty for a short time insufficiently induced NASH and inflammatory responses. On the other hand, Ty (500 mg/kg) did not have enough concentration to arouse inflammatory responses. But these conjectures are unclear and still need to be verified.

**Figure 10:**
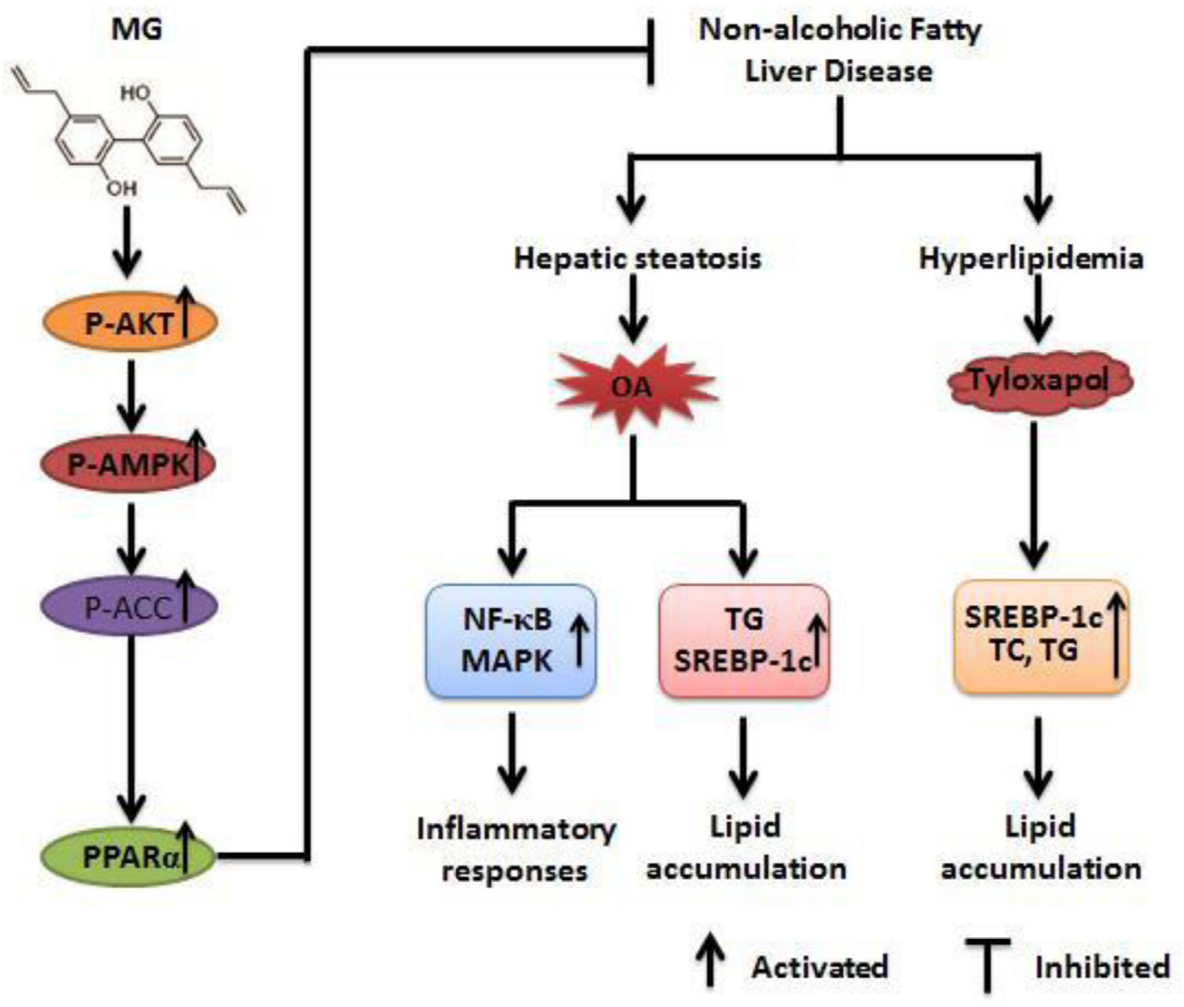
Magnolol (MG)-activated peroxisome proliferator-activated receptor α (PPARα) expression protects against inflammatory responses and lipid accumulation in oleic acid (OA)-induced steatosis of HepG2 cells and in tyloxapol-induced hyperlipidemia mice. MG can induce AMPK, AKT, and acetyl-CoA carboxylase (ACC) activation, which contribute to upregulation of PPARα expression and leads to anti-steatosis and antihyperlipidemia effects that are dependent on attenuating the induction of sterol regulatory element-binding protein 1c (SREBP-1c) protein expression. Furthermore, MG reduces OA-induced reactive oxygen species and TNF-α overproduction by inhibiting the activation of the nuclear factor-kappa B (NF-κB) and mitogen-activated protein kinase (MAPK) signaling pathways. These pathways play significant roles in repressing lipid accumulation and inflammatory responses to alleviate steatosis and hyperlipidemia.

In conclusion, as is shown in Figure 10, our results imply that MG possesses antisteatotic and antihyperlipidemic capabilities. The underlying mechanisms of MG may be inhibited by the phosphorylation of MAPK and NF-κB and activated by AKT, AMPK, and ACC. These effects are largely associated with the upregulation of PPARα and the inhibition of SREBP-1c expression. Nowadays, new therapeutic therapies are being developed to target many of signaling pathways, while most clinical trials have paid attention to monotherapy. Combination of multiple drug targets and pathogenic pathways may be most appropriate (5). Furthermore, uncovering and exploiting these mechanisms by which MG inhibits MAPK/NF-κB/SREBP-1c and activates AKT/AMPK/PPARα could yield new therapeutic approaches that could transform the outlook for patients. Taken together, the results of this study confirm that MG may be a promising candidate drug that is likely to apply as a treatment for patients with NAFLD and NASH.

## Ethics Statement

All studies were performed in accordance with the Guide for the Care and Use of Laboratory Animals published by the US National Institutes of Health. This study was reviewed and approved by the Animal Welfare and Research Ethics Committee at Jilin University.

## Author Contributions

YT conducted most of the experiments and wrote the basic manuscript. LH conducted the rest of the experiments. LW, ZL, and QZ analyzed the results. HL and BS critically revised the paper. HF and GL contributed to the experimental design process.

## Conflict of Interest Statement

The authors declare that the research was conducted in the absence of any commercial or financial relationships that could be construed as a potential conflict of interest.
